# Supplementary material for: An infectious disease model on empirical networks of human contact: bridging the gap between dynamic network data and contact matrices
Source: BMC Infect Dis. 2013 Apr 23;13:185. doi: 10.1186/1471-2334-13-185 (PMC3640968; doi:10.1186/1471-2334-13-185)
Supplement: Additional file 1 — Supporting Text. [file 1471-2334-13-185-S1.pdf]

An infectious disease model on empirical networks of human contact: bridging the gap between dynamic network data and contact matrices.
Additional file 1 - Supporting Text

A. Machens, F. Gesualdo, C. Rizzo, A.E. Tozzi, A. Barrat, C. Cattuto

1 Data and data representation

1.1 Network properties

Figure 1 displays some characteristics of the aggregated network of contacts between individuals as gathered by the SocioPatterns infrastructure. The degree of an individual in the aggregated network gives the number of distinct individuals with whom she has been in contact with at least once. The strength of a node is the sum of the durations of all the contacts that node had with other individuals. The detailed properties of the network are reported in Ref. [1].

1.2 Fitting the weight distributions for each role pair

As discussed in the main text, for each pair (X, Y) of role classes we consider the empirical cumulated duration of the contacts between all pairs of individuals x of class X and y of class Y. We used the `fitdistr` function of the R package MASS (`http://www.stats.ox.ac.uk/pub/MASS4`), which implements maximum-likelihood fitting of univariate distributions, to fit each empirical distribution with a negative binomial with parameters m and r, where m is the average of the fitted distribution and its variance is $s = m + m * m/r$.

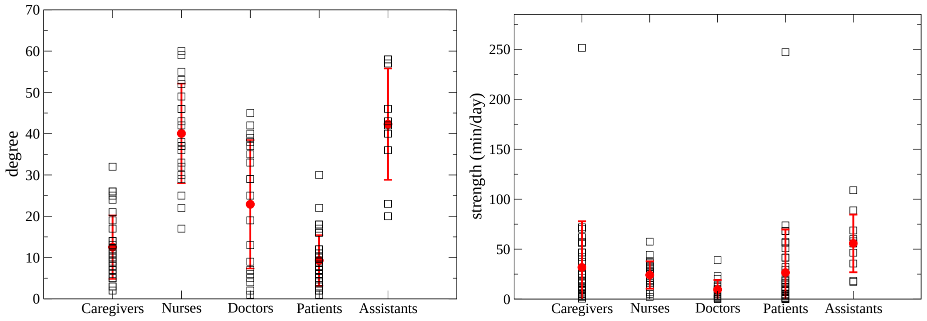

Figure 1: Left: Average degree (red dots, the red lines show the standard deviation) and actual degrees of each individual (empty squares) for each role. Right: Same for the strength, in minutes/day.

The fitting parameters are given in the main text. Figure 2 shows the comparison between the empirical and the fitted distributions.

1.3 An intermediate data representation

In the main text we described several data representations, which range from a very detailed record of the temporally-resolved contacts (DYN) to a much coarser representation in terms of a contact matrix (CM) that contains the average time spent in contact by members of given classes. The CMD (Contact Matrix of Distributions) representation takes into account the heterogeneity of contact durations between pairs of individuals who belong to a given class pair, as it is constructed by fitting the entire distribution of these durations.

Here we consider an intermediate representation between the CM and CMD ones. For each pair of roles (X, Y) we describe the distribution of weights using a bimodal distribution (instead of the negative binomial used for CMD), where values are either 0 (corresponding to an absence of link) or the average of all actual weights between individuals of roles X and Y. More in detail, let N_X and N_Y be the numbers of individuals in classes X and Y respectively, E_{XY} the number of links empirically observed between individuals of classes X and Y, and W_{XY} the the total time spent in contact by any individual of class X with any individual of class Y. Then, for each pair of individuals (x, y) with x in class X and y in class Y, x is in contact with y with probability $E_{XY}/(N_X N_Y)$, and the weight of the corresponding edge is W_{XY}/E_{XY}. With probability $1 - E_{XY}/(N_X N_Y)$, x and y are not in

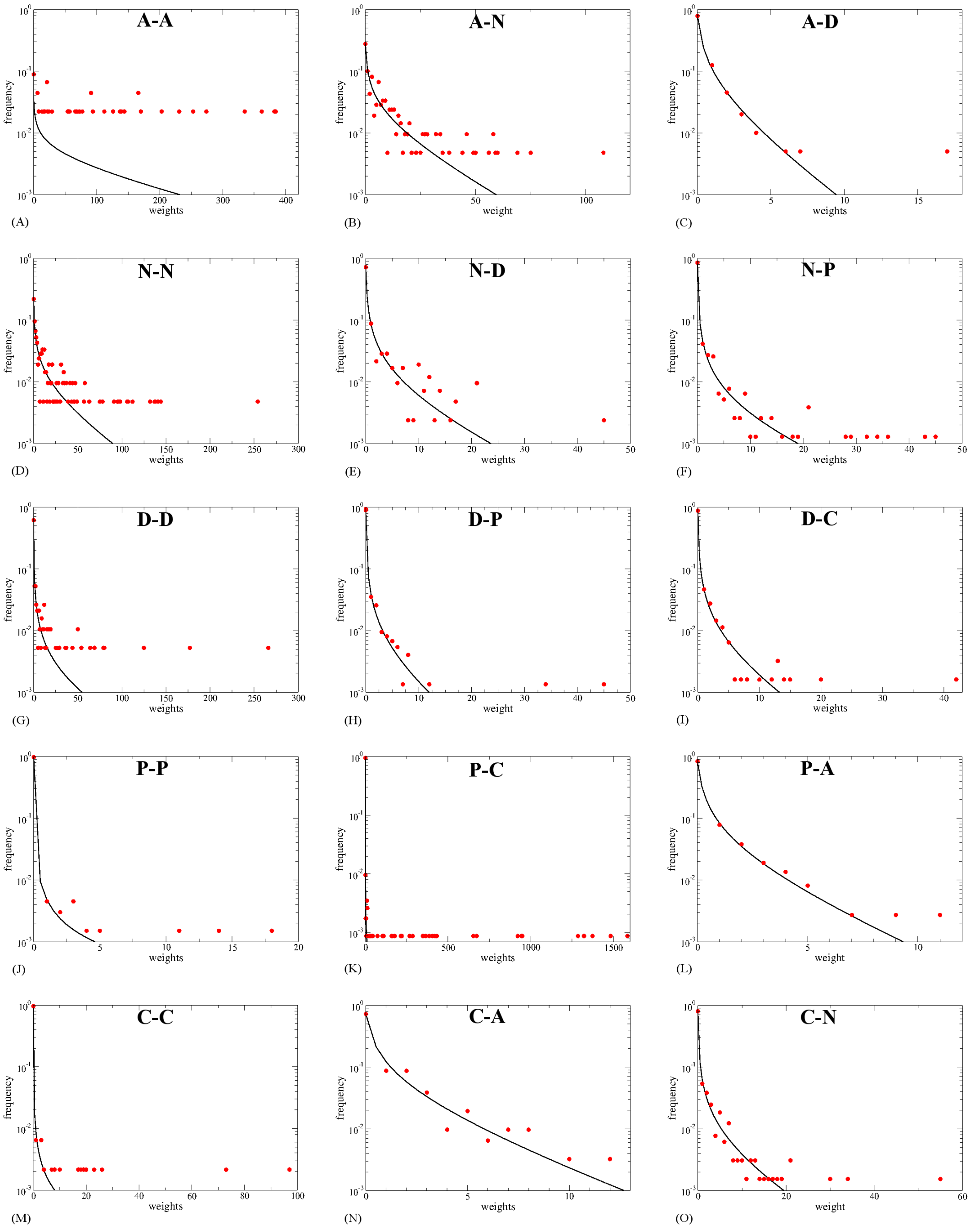

Figure 2: Weight distributions for each role pair (red dots) and negative binomial fits obtained by maximum-likelihood fitting (fitdistr function of R's MASS package). The abscissa is the weight of a link in units of 20-second intervals.

contact and the associated weight is taken as zero. Notice that for $X = Y$, $N_X N_Y$ is replaced by $N_X(N_X - 1)/2$, the correct number of within-class links. The above representation, which we call CMB (Contact Matrix of Bimodal distributions), takes into account the fact that not all pairs of individuals have been in contact, in contrast with the customary CM representation.

1.4 Properties of the different data representations

Tables 1 and 2 report some properties of the weights and topology of the observed contact patterns according to the various data representations we consider. The CMB, CMD and HOM representations all capture the main topological properties of the HET network, as well as the average link weight, while the CMD representation is the only one that accounts for the large dispersion in the cumulated durations of the contacts.

Data representation	$\langle w \rangle$ ($\times 10^{-4}$)	σ_w ($\times 10^{-5}$)	W	w_{max} ($\times 10^{-4}$)
Fully connected	1.59	0.00	1.12	1.6
CM	1.59	0.01	1.12	34.8
CMB	9.11	0.49	1.12	99.8
CMD	9.01	3.75	1.11	1348.9
HOM	9.11	0.00	1.12	9.1
HET	9.11	3.47	1.12	1710.3

Table 1: Average weight $\langle w \rangle$, weight variance σ_w, sum of the weights W and maximum weight w_{max} of the weight distributions. The average and variance are computed on the non-zero weights only.

Data representation	N_0	$\langle d \rangle$	σ_d	$\langle C \rangle$
Fully connected	0	118.0	0.0	1.00
CM	0	118.0	0.0	1.00
CMB	5794	20.6	176.4	0.34
CMD	5789	20.7	178.2	0.35
HOM	5794	20.6	267.1	0.53
HET	5794	20.6	267.1	0.53

Table 2: Number N_0 of zero-weight links, average degree $\langle d \rangle$, degree variance σ_d, and average clustering coefficient $\langle C \rangle$ of each network representation.

1.5 Rescaling the rate of infection to compare the spread over different data representations

As the probability of disease transmission between an infectious and a susceptible individual depends on the time spent in contact, in order to meaningfully compare the evolution of spreading processes over dynamical and static networks, it is necessary to rescale adequately the rate of infection β (see also the discussion in Ref. [2]). Let us consider the contact between an infectious individual A and a susceptible individual B: the probability that the infection is transmitted from A to B during a time interval dt is βdt. On representing contact patterns by means of a static weighted network (e.g., the HOM/HET cases) in which the weight W_{AB} of the link between A and B represents the total duration of the contacts between those nodes, the infection probability over the interval dt needs to be set as $\beta W_{AB} dt/\Delta T$, where ΔT is the total temporal span of the data set, so that we obtain the same average infection probability in all networks.

2 Simulation of epidemic spread

2.1 Additional measures

In the main text we focused on the probability of extinction and on the attack rate, as these quantities are the most important to quantify the impact of a disease and to assess the effectiveness of interventions. Other properties can also be measured and can help to assess the differences among the simulated epidemic dynamics based on the different data representations. In particular, here we consider the peak time of the epidemic, the duration of the epidemic, and the respective distributions of these quantities over an ensemble of stochastic realizations of the spreading dynamics.

We also consider the issue of the reproductive number R_0, defined as the expected number of secondary infections from an initial infected individual in a completely susceptible host population [3]. Several methods can be used to compute this quantity [4, 5] (40, 41), possibly yielding different estimates [6] for the same epidemiological parameters.

Similarly to other works [7, 8], we compute here the number of secondary cases from each single initial randomly chosen infectious individual, and obtain the distribution of these numbers S over an ensemble of stochastic

realizations of the dynamics. The basic reproductive number is then approximated by the average $\langle S \rangle$ of the number of secondary cases over this distribution.

2.2 Simulated spread on the fully connected network and on the CMB data representation

Figure 3 shows the distributions of the fraction of the final number of cases for various contact pattern representations, for the same spreading parameters used in the main text. The HET, CM and CMD cases are the same as in Figure 2A of the main text. The fully connected case yields a very strong overestimation of the final number of cases and a very small probability of having a final attack rate lower than 10%. The CMB representation, as expected, yields an intermediate result between CMD and CM, which remains very different from the result of a epidemic spread on the HET network.

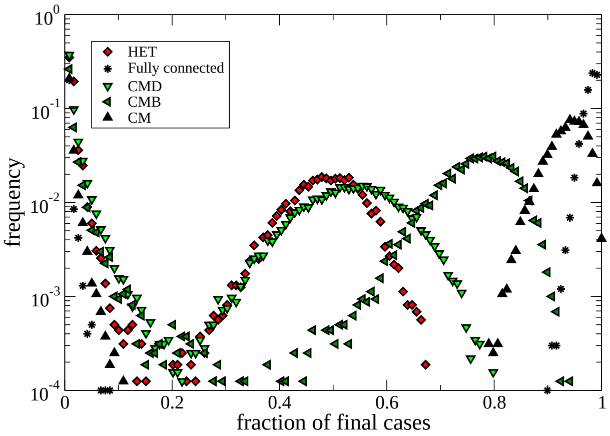

Figure 3: Distribution of the fraction of the final number of cases for various contact pattern representations for $\beta = 2.8 \times 10^{-3} s^{-1}$, $1/\sigma = 0.5$ days, $1/\nu = 1$day.

2.3 Reproductive number and epidemic peak and ending times

Figure 4 reports the distributions of the number of secondary cases from the initial seed, averaged over the initial seed of the epidemic and over the

Data representation	$\langle S \rangle$
DYN	1.06
HET	1.66
HOM	3.71
CM	3.87
CMD	1.67

Table 3: Average number $\langle S \rangle$ of secondary infections from an initial infected individual in a completely susceptible host population. The values of $\langle S \rangle$ are computed as averages of the distributions shown in Figure 4.

stochastic realizations of epidemic spread, for various contact pattern representations. The most probable number of secondary cases is zero in all cases, and the distribution has a typical exponential shape, ranging up to values of $20-30$ individuals. The distributions are slightly broader for the HOM and CM representations.

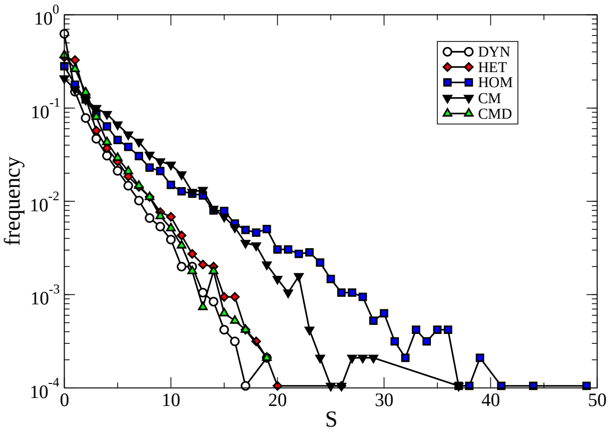

Figure 4: Distribution of the number of secondary cases from each single initial randomly chosen infectious individual, for the different contact pattern representations, with parameters $\beta = 2.8 \times 10^{-3} s^{-1}$, $1/\sigma = 0.5$ days, $1/\nu =$ 1day.

Table 3 gives the value of the average number of secondary cases $\langle S \rangle$ for each case. We notice that while Ref. [2] reported similar average values for the DYN and HET cases for the case of a spread in an unstructured population, here we observe a smaller value for the DYN network. For the first set of

parameters of the SEIR model, corresponding to slower disease propagation, the difference between the values for DYN and HET (not shown) is smaller. It is important to notice that for both parameter sets, the HET and CMD representations yield very close values of $\langle S \rangle$, while the HOM and CM cases both yield higher values. This is remarkable because the contact matrix of distributions (CMD) is a compact representation that is not individual-based and does not preserve the topology of the empirical contact network, which is instead preserved by the finer-grained HET contact network.

Finally, Figure 5 shows the distributions of the peak and end times of the epidemic. Although the distribution is slightly more peaked at earlier times for the HOM case, no strong differences are observed between the different cases. Each representation is thus able to yield a good estimation of the epidemic timing.

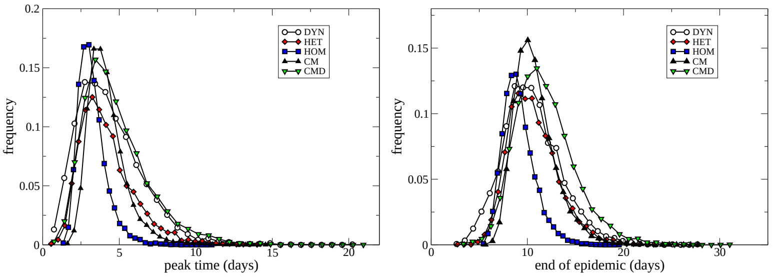

Figure 5: Distributions of the peak times (left) and end times (right) of the simulated epidemic, for each representation, and restricted to the runs with an attack rate higher than 10%. Parameters values are $\beta = 2.8 \times 10^{-3} s^{-1}$, $1/\sigma = 0.5$ days, and $1/\nu = 1$day.

3 Results of the spreading simulations for the second set of parameters of the SEIR model

In order to assess the robustness of our results we have considered, as mentioned in the main text, two different sets of parameters for the SEIR model. Here we report the results of spreading simulations with $\beta = 6.9 \times 10^{-4} s^{-1}$, $1/\sigma = 1$ day, and $1/\nu = 2$days on the different representations of contact patterns. This set of parameters corresponds to a slower propagation with respect to the one discussed in the main text.

Figure 6, similarly to Figure 2A of the main text, reports the global distribution of the fraction of the final number of cases, averaged over all possible seeds, for the various contact patterns representations. The results are very similar to the ones obtained with the other set of parameters, except that the results of the DYN, HET and CMD are even closer. All the other representations lead to a clear underestimation of the probability of having a small final attack rate and, in the case of a large attack rate, to a strong overestimation of the number of final cases (see Table 4). Table 5 breaks down these results according to the role class of the initial seed, and Figure 7 shows the distributions of the final attack rates within each class. Once again, at this very detailed level the similarity between the results for DYN, HET and CMD is clear, while the CM and HOM representations do not capture correctly the relative risks of the various classes.

Finally, Table 4 shows that the peak time and end time of the epidemic are reasonably well approximated even by the coarser representations.

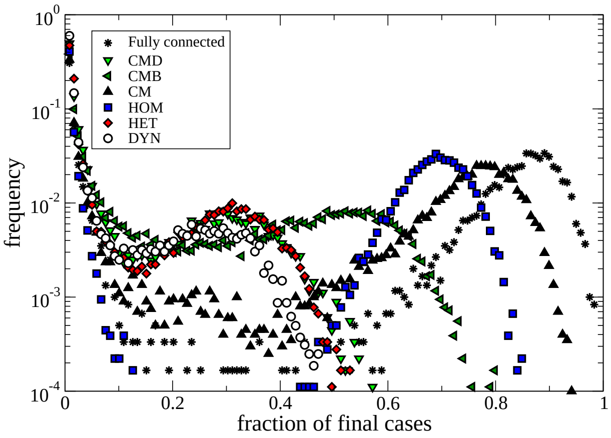

Figure 6: Distribution of the fraction of the final number of cases for the various contact pattern representations for $\beta = 6.9 \times 10^{-4} s^{-1}$, $1/\sigma = 1$ day, $1/\nu = 2$days.

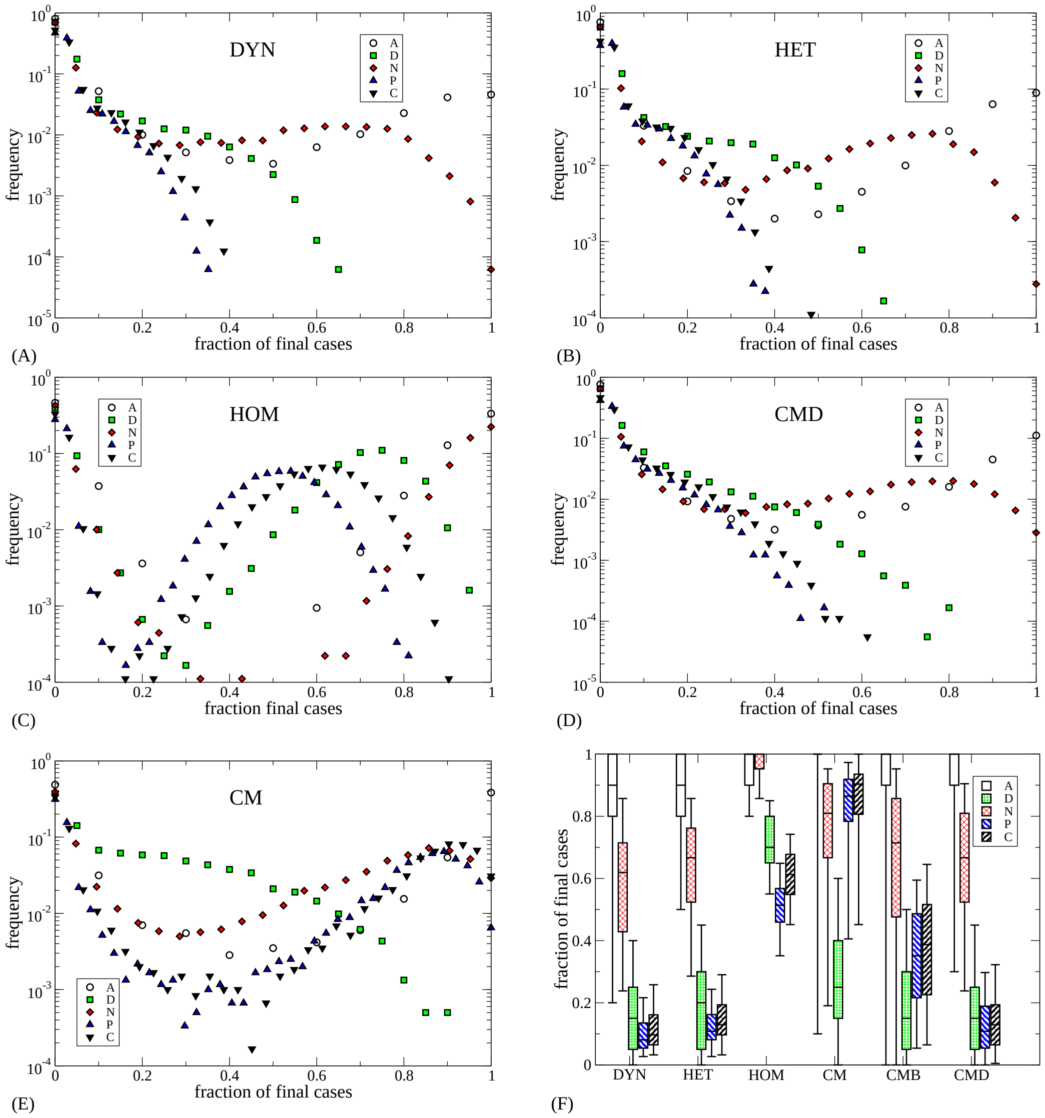

Figure 7: A)-E) Distributions of the fraction of individuals of each class reached by the epidemic for various contact patterns. F) Boxplots corresponding to these distributions when the global attack rate is larger than 10%. Here $\beta = 6.9 \times 10^{-4} s^{-1}$, $1/\sigma = 1$day, and $1/\nu = 2$ days.

Data representation	final size	peak time	end time
DYN	31(10)	8.4(4.5)	19.7(6.4)
HET	35(10)	8.9(4.6)	20.7(6.2)
HOM	82(7)	9.3(3.1)	22.3(4.7)
CM	86(17)	13.0(5.5)	28.9(7.5)
CMB	50(19)	11.4(6.1)	25.7(8.5)
CMD	35(11)	9.5(5.0)	21.9(7.1)

Table 4: Summary of the average properties of the runs leading to a final attack rate (AR) higher than 10%, for $\beta = 6.9 \times 10^{-4} s^{-1}$, $1/\sigma = 1$day, and $1/\nu = 2$days. The standard deviation is given in parentheses.

	DYN		HET		HOM		CM		CMB		CMD	
seed	EP	AR10	EP	AR10	EP	AR10	EP	AR10	EP	AR10	EP	AR10
All	0.60	0.86	0.47	0.80	0.41	0.50	0.33	0.48	0.38	0.65	0.50	0.80
A	0.35	0.51	0.21	0.34	0.20	0.27	0.18	0.26	0.18	0.28	0.20	0.33
D	0.71	0.93	0.65	0.87	0.40	0.49	0.56	0.81	0.57	0.84	0.59	0.91
N	0.50	0.70	0.39	0.56	0.19	0.28	0.33	0.48	0.33	0.52	0.36	0.58
P	0.66	0.95	0.51	0.91	0.54	0.64	0.30	0.44	0.40	0.71	0.58	0.92
C	0.59	0.95	0.45	0.91	0.47	0.58	0.27	0.40	0.35	0.68	0.52	0.90

Table 5: Extinction probability (EP) and fraction of runs leading to an attack rate of at most 10% (AR10), for the various contact pattern representations and as a function of the role class of the seed. Parameter values are $\beta = 6.9 \times 10^{-4} s^{-1}$, $1/\sigma = 1$day, and $1/\nu = 2$days.

4 Daily aggregated networks and contact matrices

As mentioned in the main text, based on the dynamical network that describes the contact patterns in the ward of an hospital over a 1-week interval, we have build static networks by aggregating all the interactions that takes place during the course of the week. Therefore, all of the data representations we considered assume that all invididuals are present at all times, and that the contact patterns are the same every day.

In Ref. [2], on the other hand, which focuses on contact patterns during a 2-day conference, two different daily networks were constructed. In this case, the epidemic spreading simulations performed over the DYN and HET networks yielded almost indistinguishable results.

Therefore, here we also consider a set of data representations aggregated on the daily scale: for each day we aggregate the contacts recorded during that day and we construct daily HET, HOM, CM, CMD representations. These representations take into account the fact that not all individuals are present each day, and that the patterns of contacts may be different from day to day.

As shown in Figure 8, the SEIR spreading simulations performed on the various data representations yield results that are only slightly different from the ones obtained when the dynamical data is aggregated at the weekly scale. The results obtained for the HET representation become even closer to the ones of the DYN case, especially for the first set of SEIR parameters, in agreement with the results of Ref. [2]. In the case of the CMD representation, the results do not change significantly. Moreover, the results obtained with the HOM and CM representations are still very far from the ones based on the DYN, HET and CMD representations, despite the additional information at the daily scale. Thus, these results highlight the interesting properties of the CMD representation, which, despite using only very parsimonious information, yields results that are very close to the case of the full dynamical representation.

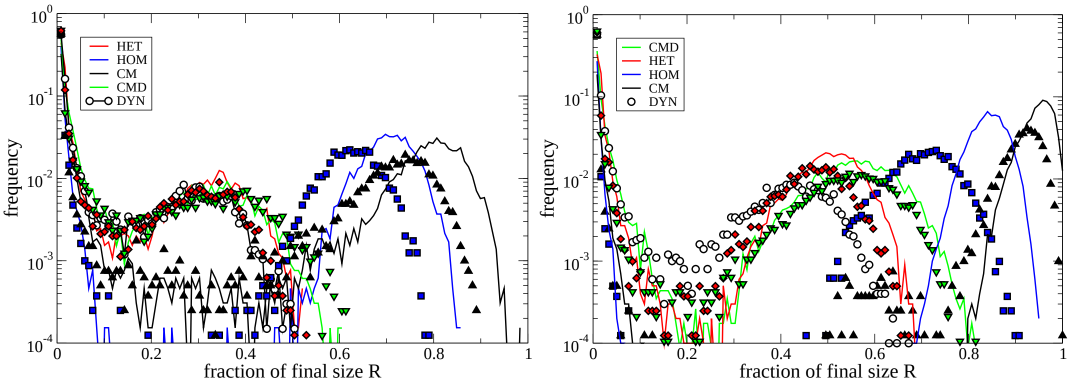

Figure 8: Distribution of the fraction of final cases for the various data representations, comparing the cases in which data is aggregated on the whole week (lines) and the cases in which daily networks or matrices are used (symbols). Left: $\beta = 2.8 \times 10^{-3} s^{-1}$, $1/\sigma = 0.5$ days, $1/\nu = 1$day; Right: $\beta = p * 6.9 \times 10^{-4} s{-}1$, $1/\sigma = 1$ day, $1/\nu = 2$days.

5 Epidemic spreading simulations for fixed $\langle S \rangle$

Figure 4 and Table 3 show that the reproductive number R_0, approximated by the average $\langle S \rangle$ over different realizations of the number of secondary cases from one randomly chosen initial infectious individual, is strongly overestimated when using the HOM and CM representation of contact patterns. One could thus ask whether the discrepancies between the results obtained by spreading simulations on the HOM and CM contact patterns on the one hand, and on the HET network on the other hand, may be simply due to differences in $\langle S \rangle$. To check this point, here we study the dynamics of spreading processes *at fixed* $\langle S \rangle$.

5.1 Procedure

We simulate an SEIR spreading process for the HOM and CM representations, calibrating the spreading probability with a rescaling factor p, so that the spreading probability per unit time is $p\beta$. We compute the resulting $\langle S \rangle$ as a function of p, as shown in Figure 9. We can thus calibrate the SEIR parameters by choosing rescaling factors p_{HOM} and p_{CM} that lead to the

same $\langle S \rangle$ measured for the HET network. We call the corresponding cases HOM(c) and CM(c).

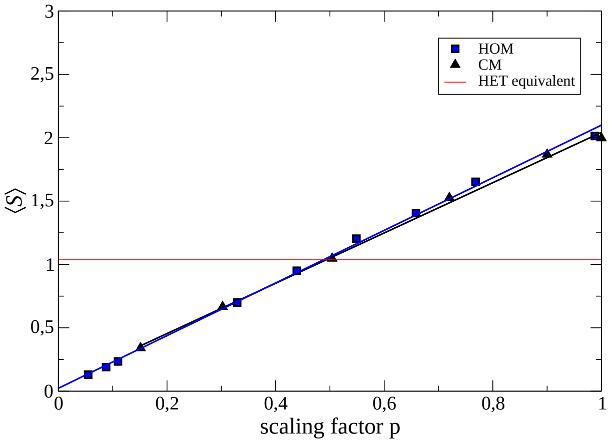

Figure 9: Average number of secondary cases $\langle S \rangle$ for the HOM and CM contact patterns, for $\beta = p * 6.9 \times 10^{-4} s^{-1}$, $1/\sigma = 1$ day, $1/\nu = 2$days. For $p = 0.488$, $\langle S \rangle$ of the HOM network is equivalent to the $\langle S \rangle$ of the HET network (with $p = 1$). For CM the corresponding value is $p = 0.494$.

5.2 Results

Figures 10, 11, 12, and Tables 6 and 7 report the results of the spreading simulations performed on the HOM and CM contact patterns with calibrated spreading parameter, so that the resulting value of $\langle S \rangle$ is the same as in the case of the HET network.

In this case, although the results are closer to each other when the calibration (rescaling factor p) is not carried out, the attack rate is still overestimated (in particular for the HOM case) and the relative attack rates within the various classes are still not correctly accounted for, especially for CM(c).

References

[1] L. Isella, M. Romano, A. Barrat, C. Cattuto, V. Colizza, W. Van den Broeck, F. Gesualdo, E. Pandolfi, L. Rava, C. Rizzo, A.E. Tozzi. Close encounters in a pediatric ward: measuring face-to-face proximity and mixing patterns with wearable sensors. PLoS ONE **6**(2): e17144 (2011)

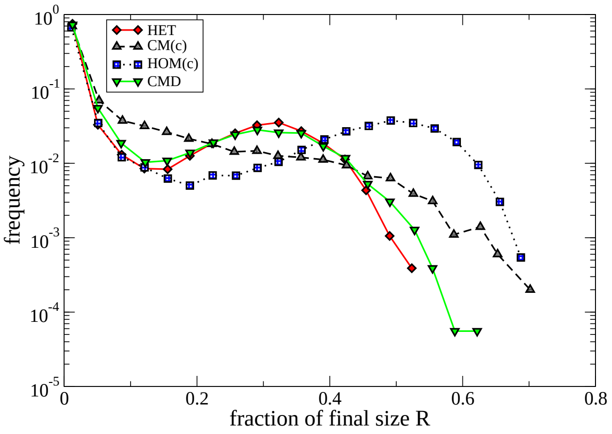

Figure 10: Distribution of the final fraction of cases for the contact pattern representations with calibrated spreading parameter: $\beta = p \times 6.9 \times 10^{-4} s^{-1}$, $1/\sigma = 1$day, $1/\nu = 2$days. For comparison we also show the results for the HET and CMD cases with $\beta = 6.9 \times 10^{-4} s^{-1}$, $1/\sigma = 1$day, $1/\nu = 2$days.

	CM(c)		HOM(c)	
seed	EP	AR10	EP	AR10
All	0.50	0.80	0.54	0.72
A	0.29	0.60	0.31	0.52
D	0.73	0.97	0.51	0.71
N	0.50	0.82	0.31	0.51
P	0.48	0.79	0.68	0.84
C	0.45	0.78	0.61	0.78

Table 6: Extinction probability (EP) and fraction of runs leading to a final AR of at most 10% (AR10) in the calibrated contact pattern representations and as a function of the class of the seed. Parameter values are $\beta = 6.9 \times 10^{-4} s^{-1}$, $1/\sigma = 1$day, and $1/\nu = 2$ days.

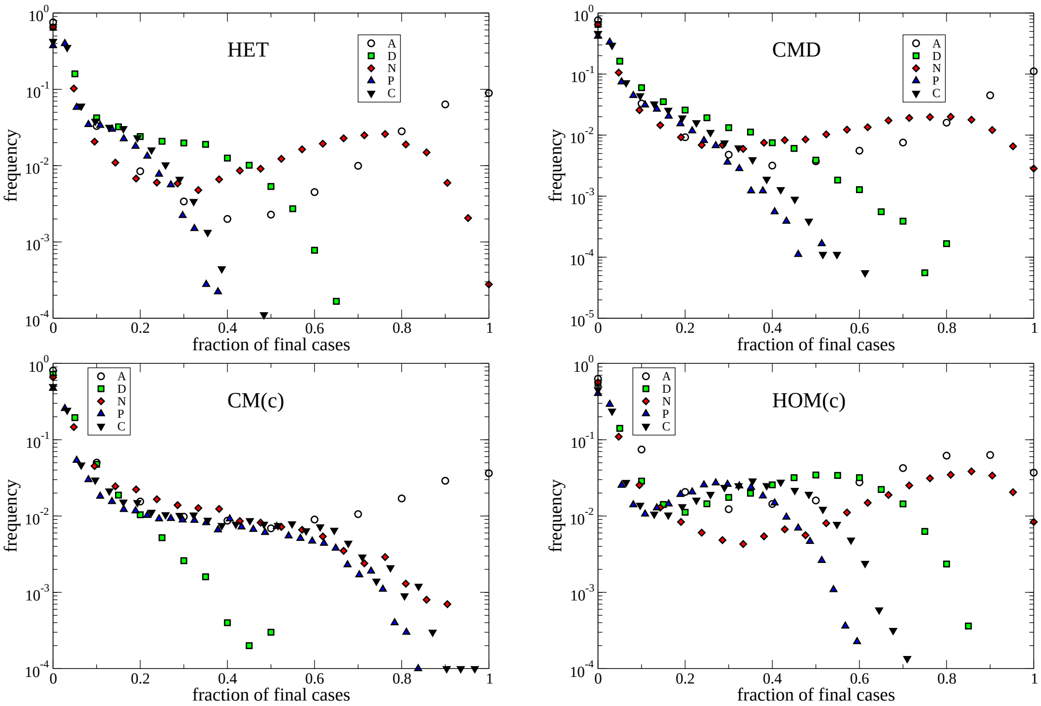

Figure 11: Distributions of the fraction of individuals of each class reached by the epidemic spread for the calibrated HOM and CM contact patterns ($\beta = p \times 6.9 \times 10^{-4} s^{-1}$, $1/\sigma$ = 1day, $1/\nu$ = 2days), compared to the HET and CMD cases ($\beta = 6.9 \times 10^{-4} s^{-1}$, $1/\sigma$ = 1day, $1/\nu$ = 2days).

Contact Pattern	final size	peak time	end time
DYN	31(10)	8.4(4.5)	19.7(6.4)
HET	35(10)	8.9(4.6)	20.7(6.2)
HOM	82(7)	9.3(3.1)	22.3(4.7)
CM	86(17)	13.0(5.5)	28.9(7.5)
CMD	35(11)	9.5(5.0)	21.9(7.1)
HOM(c)	53(15)	11.5(5.3)	25.2(7.2)
CM(c)	32(15)	11.0(6.8)	24.2(9.4)

Table 7: Summary of the average properties of the realizations that lead to a final AR higher than 10%, for $\beta = 6.9 \times 10^{-4} s^{-1}$, $1/\sigma$ = 1day, and $1/\nu = 2$ days.

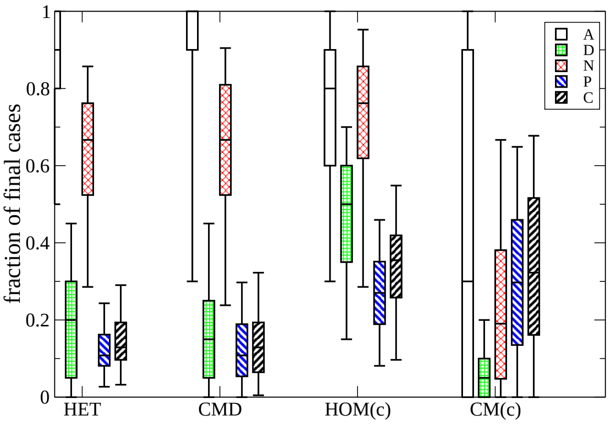

Figure 12: Boxplots showing the distributions of Fig. 11 when the final attack rate is larger than 10%, for the HOM and CM representations with calibrated parameters ($\beta = p \times 6.9 \times 10^{-4} s^{-1}$, $1/\sigma = 1$day, $1/\nu = 2$ days) and for the HET and CMD cases ($\beta = 6.9 \times 10^{-4} s^{-1}$, $1/\sigma = 1$day, $1/\nu = 2$ days).

[2] J. Stehlé, N. Voirin, A. Barrat, C. Cattuto, V. Colizza, L. Isella, C. Régis, J.-F. Pinton, N. Khanafer, W. Van den Broeck, P. Vanhems. Simulation of a SEIR infectious disease model on the dynamic contact network of conference attendees. BMC Medicine **9**:87 (2011)

[3] R.M. Anderson, R.M. May. Infectious Diseases of Humans: dynamics and control. Oxford University Press (1991).

[4] O. Diekmann, J. Heersterbeek, J. Metz. On the definition and the computation of the basic reproduction number ratio R0 in models for infectious diseases in heterogeneous populations. J Math Biol **28**:365-82 (1990).

[5] J.M. Heffernan, R.J. Smith, L.M.Wahl. Perspectives on the basic reproductive ratio. J R Soc Interface **2**:281-93 (2005).

[6] R. Breban, R. Vardavas, S. Blower. Theory versus data: how to calculate R0? PloS One **2**:e282 (2007).

[7] T.C. Germann, K. Kadau, I.M. Longini Jr, C.A. Macken. Mitigation strategies for pandemic influenza in the United States. Proc Natl Acad Sci USA **103**:5935 (2006).

[8] M. Ajelli, S. Merler. The Impact of the Unstructured Contacts Component in Influenza Pandemic Modeling. PLoS ONE **3**: e1519 (2008).
